# Posterior reversible encephalopathy syndrome after anlotinib treatment for small cell lung cancer: A case report and literature review

**DOI:** 10.3389/fphar.2023.1126235

**Published:** 2023-02-06

**Authors:** Xiaomeng Zou, Peng Zhou, Wei Lv, Chuanyong Liu, Jie Liu

**Affiliations:** ^1^ Department of Clinical Medical College, Weifang Medical University, Weifang, China; ^2^ Department of Oncology, Central Hospital Affiliated to Shandong First Medical University, Jinan, China; ^3^ Department of Medical Imaging Center, Central Hospital Affiliated to Shandong First Medical University, Jinan, China; ^4^ Department of Cardiology, Central Hospital Affiliated to Shandong First Medical University, Jinan, China

**Keywords:** posterior reversible encephalopathy syndrome, anlotinib, antiangiogenic therapy, small cell lung cancer, case report

## Abstract

Anlotinib is an oral multi-targeted tyrosine kinase inhibitor as a third-line and subsequent treatment for patients with small cell lung cancer (SCLC) in China. The neurotoxicity is less reported. Posterior reversible encephalopathy syndrome (PRES) is characterized by headaches, seizures, encephalopathy, and visual disturbances, as well as focal reversible vasogenic edema seen on neuroimages. Here, we presented a case of PRES in a small cell lung cancer (SCLC) patient associated with anlotinib. A 37-year-old female patient, who had a history of diabetes, with extensive-stage SCLC received anlotinib after third-line chemotherapy. Ten cycles of anlotinib later, the patient experienced visual disturbance and was diagnosed with PRES based on the typical demyelination of white matter obtained in the brain magnetic resonance. During anlotinib therapy, the patient did not develop anti-VEGF therapy-induced hypertension. Subsequently, the patient stopped anlotinib, but she did not recover from symptoms. We also summarized the characteristics of fifty-four cases of PRES caused by antiangiogenic drugs in the literature. Based on our experience and the literature review, the incidence of PRES induced by antiangiogenic drugs is low, and the symptom can resolve upon stopping the medications. However, some cases still have a poor prognosis and the underlying mechanism requires further investigation. In addition, early detection and treatment of PRES are essential for physicians.

## Introduction

Small cell lung cancer (SCLC) accounts for approximately 15% of all newly diagnosed lung cancer cases and about 60%–70% of patients present with extensive-stage disease (ES-SCLC) at the initial visit (Siegel et al., 2017; Huang et al., 2021). Progress in the treatment of ES-SCLC over the past 30 years has been modest. Chemotherapy with platinum (cisplatin or carboplatin) plus etoposide is the standard first-line therapy for patients with ES-SCLC (Zugazagoitia and Paz-Ares, 2022). To date, topotecan is the only Food and Drug Administration (FDA) approved second-line drug for the treatment of recurrent metastatic SCLC (Ganti et al., 2021). Currently, there was no standard third-line therapy recommended in guidelines (Dingemans et al., 2021; Ganti et al., 2021).

Anlotinib hydrochloride is an oral multi-targeted tyrosine kinase inhibitor of vascular endothelial growth factor receptor (VEGFR)-1/2/3, fibroblast growth factor receptor (FGFR)-1-4, platelet-derived growth factor receptor-α/β (PDGFR-α/β) and c-kit-proto-oncogene-protein (c-Kit). Cheng et al. launched a Phase 2 trial to evaluate the efficacy and safety of anlotinib as a third-line and subsequent treatment for patients with SCLC in China (ALTER 1202) and found that anlotinib showed improved PFS and OS than placebo with favorable safety profile (Cheng et al., 2021). In this trial, the most common adverse events (AEs) of anlotinib were hypertension, fatigue, thyroid-stimulating hormone elevation, anorexia, hypertriglyceridemia, hand-foot syndrome, and hypercholesterolemia (Cheng et al., 2021). And the most common grade 3 or higher AEs were hypertension (11 [13.6%]), gamma-glutamyl transpeptidase elevation (4 [4.9%]), and hand and foot skin reaction (4 [4.9%]) (Cheng et al., 2021). Similar results were seen in the ALTER0303, ALTER 0703, ALTER 01031 and a double-blind randomized phase 2 trials (Cheng et al., 2021; Chi et al., 2021; Huang et al., 2021; Huang et al., 2021; Li et al., 2021). However, the neurotoxicity was less reported. The most common AEs related to nervous system disorder was reported to be headache (33 [11.2%]) in the ALTER0303 trial (Han et al., 2018). In addition, there were a few rare AEs reported such as insomnia, sensory neurotoxicity, and seizure (Huang et al., 2021; Liu et al., 2021; Lu et al., 2022; Nie et al., 2022; Shao et al., 2022; Zou et al., 2022). Although a few cases of posterior reversible encephalopathy syndrome (PRES) caused by some anti-angiogenic drugs were reported in the literature (Koopman et al., 2008; Chelis et al., 2012; Myint et al., 2014; Sharma et al., 2016; Saraceno et al., 2017; Miaris et al., 2019; Van Pelt et al., 2020), no PRES has been previously reported in SCLC patients treated with anlotinib.

Here, we present a case with ES-SCLC who achieved a significant progression-free survival benefit from anlotinib as fourth-line therapy, report the PRES during anlotinib treatment, and review the characteristics of PRES induced by antiangiogenic therapy.

## Case presentation

**FIGURE 1:**
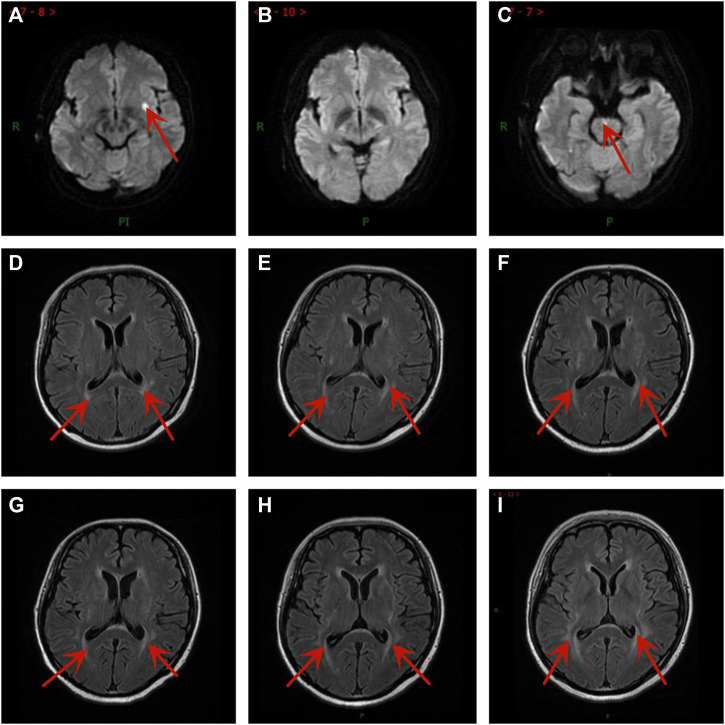
Brain magnetic resonance imaging images at different time points. **(A)** Brain metastases were newly diagnosed after second-line treatment. **(B)** One month after brain radiation therapy. **(C)** New brain metastasis after taking anlotinib 10 cycles. The PRES developed gradually after taking anlotinib for **(D)** 1 cycle, **(E)** 3 cycles, **(F)** 4 cycles, **(G)** 6 cycles, **(H)** 8 cycles, and **(I)** 10 cycles.

A 37-year-old Chinese woman was admitted to the hospital with repeated cough in February 2019. The patient had a 3-year history of diabetes and no smoking history. Chest computed tomography (CT) scan revealed a mass in the lower lobe of the left lung. Fiberoptic bronchoscopy revealed a tumor in the left lower lobe. Pathologic examination of the biopsies showed SCLC. Positron emission tomography (PET)/CT scan showed a huge soft tissue mass in the lower lobe of the left lung and left hilar nodule metastasis. Accordingly, she was diagnosed with left lung SCLC with limited-stage (LS-SCLC) based on the Veterans Administration Lung Study Group (VALSG) 2-stage classification scheme (Kalemkerian and Gadgeel, 2013). Subsequently, she received first-line treatment with etoposide/cisplatin (EP) for four cycles plus definitive thoracic radiotherapy (RT). However, 1 month after the completion of initial therapy, abdominal CT revealed right adrenal metastasis. Then she received four cycles of irinotecan as the second-line treatment from November 2019 to January 2020 and followed by radiation therapy for right adrenal metastasis. On 29 March 2020, her brain magnetic resonance imaging (MRI) showed the left basal ganglia metastasis (Figure 1A). Meanwhile, the abdominal CT scan discovered left adrenal metastasis. And she was diagnosed with hyponatremia (121 mmol/L) and the serum neuron-specific enolase (NSE) (45.1 ng/mL) level was higher than the normal range. Then she received whole-brain radiation therapy (WBRT) of a total dose of 37.5 Gy (in 15 fractions), which had achieved radiographic complete regression (Figure 1B) and left adrenal gland radiation of a total dose of 46.0 Gy (in 23 fractions) from 9 April to 11 May 2020. At the same time, she received two cycles of chemotherapy with cyclophosphamide/adriamycin/cisplatin (CAP).

**FIGURE 2:**
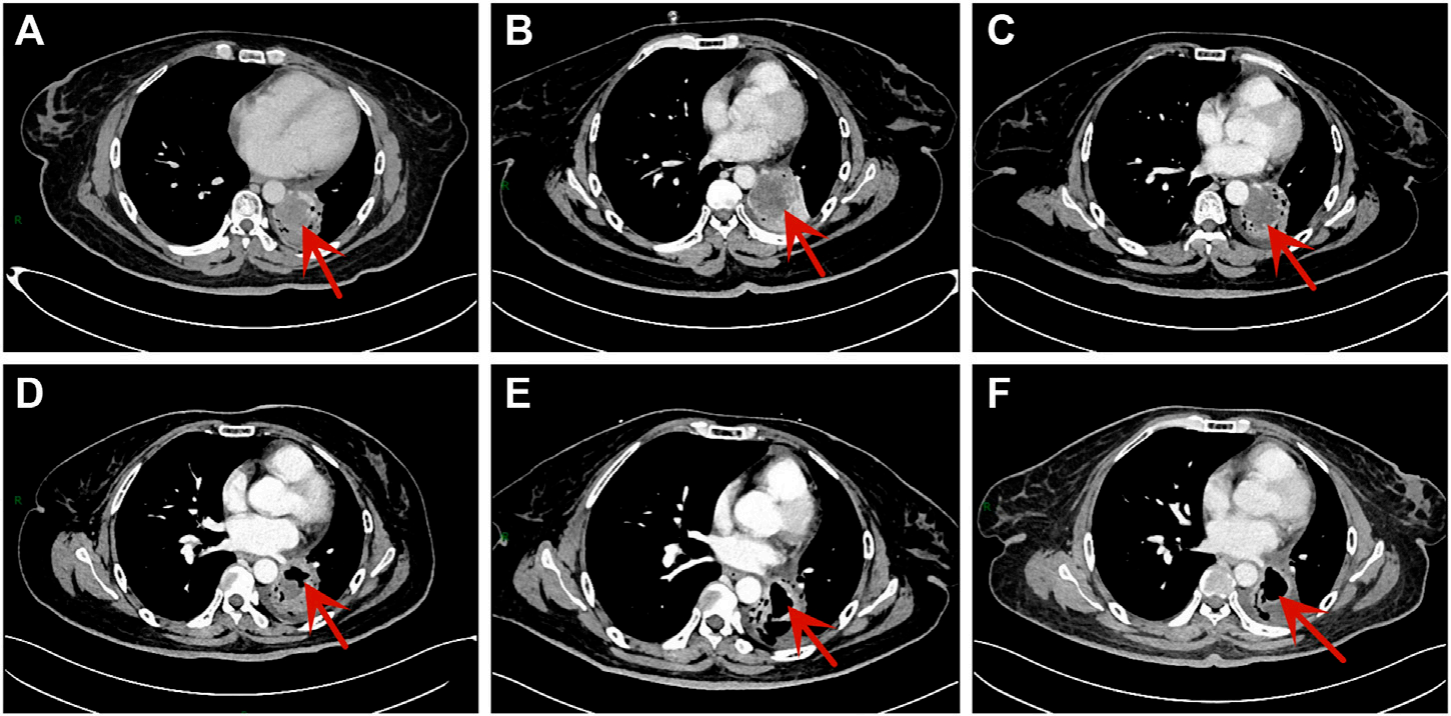
Chest CT scans of the patient. **(A)** The scan was performed after second-line treatment. **(B)** The scan was performed after third-line treatment. **(C)** The scan was performed after taking anlotinib for one cycle. **(D)**The scan showed tumor cavity formation after taking anlotinib for six cycles. **(E)** The scan showed tumor cavity was larger after taking anlotinib for eight cycles. **(F)** The tumor cavity didn’t change significantly after taking anlotinib for 10 cycles.

However, the patient complained about increasing cough with dyspnea on exertion and fatigue. The contrast-enhanced CT (10 June 2020) showed a 46 mm × 39 mm mass in the left lower lobe, which was larger than before (Figures 2A,B). No evidence of metastasis was found by the brain MRI. In addition, the serum NSE (35.4 ng/mL) level was also elevated. According to Response Evaluation Criteria in Solid Tumors version 1.1 (RECIST 1.1), efficacy assessment was progression of disease (PD). Subsequently, we administered anlotinib (10 mg/day once daily orally, 2 weeks on and 1 week off) as the fourth-line treatment on 18 June 2020 according to the result of ALTER 1202 (Cheng et al., 2021).

After one cycle of anlotinib, we performed the first evaluation by CT and brain MRI, which showed a stable disease (SD) (Figure 2C). Simultaneously, not only the level of NSE (21.2 ng/mL) declined rapidly, but also the sodium level was restored to normal. Then the efficacy was evaluated once every two cycles. After six cycles, she had a reduced cough with her Eastern Cooperative Oncology Group (ECOG) performance status ≤1 point. Additionally, the serum sodium and NSE level remained normal. Chest CT showed tumor cavity formation and tumor assessment was SD (Figures 2D, E, F).

During the treatment with anlotinib (10 mg), the most severe adverse effect was oral ulcer, which was evaluated as graded 3 according to National Cancer Institute Common Toxicity Criteria for Adverse Events (NCI-CTCAE, version 5.0). As a result, we reduced the dose of anlotinib from 10mg to 8 mg on 18 November 2020. Her brain MRI showed mild demyelination of white matter after one cycle with anlotinib and the extension of the demyelinating lesions increased slowly (Figures 1D–I), but she had no neurological symptoms. Encouraged by the result, the treatment was not interrupted.

After taking anlotinib for 7.5 months, the patient suffered from loss of appetite, weight loss, excessive fatigue, and visual disturbance. The enhanced CT scan showed multiple metastatic lesions in the bilateral ovaries on 2 February 2021. Her contrast-enhanced MRI showed the demyelination of white matter in T2-weighted fluid attenuated inversion recovery (FLAIR) images and the pons had enhancement lesions, which was considered metastasis (Figure 1C). We thought the visual disturbance was related to PRES, not brain metastases. The tumor assessment was PD. So anlotinib was discontinued. During the anlotinib therapy, the patient did not develop anti-VEGF therapy-induced hypertension. Two months later, the blurred vision was not restored, and there were no significant changes in her brain MRI. In conclusion, she gained a progression-free survival (PFS) of 7.5 months. Later, the patient had received a clinical trial and the best supportive treatment. Sadly, she passed away on 14 January 2022.

## Discussion

PRES, also known as Reversible posterior leukoencephalopathy syndrome (RPLS), is a brain-capillary leak syndrome related to hypertension, fluid retention, and the cytotoxic effects of immunosuppressive agents on the vascular endothelium, characterized by seizures, headache, altered mental status, and visual disturbances (Mergen et al., 2021). At present, the diagnosis of PRES mainly relies on MRI findings (Lee et al., 2012), characterized by white matter vasogenic edema affecting the brain’s posterior occipital and parietal lobes (Fugate and Rabinstein, 2015). Classical MRI findings include high signal intensity on T2-weighted and FLAIR images, predominantly in the posterior regions, particularly parieto-occipital lobes (Lee et al., 2012). However, there were PRES cases also occurring in the frontal lobes, basal ganglia, thalamus, and brainstem (Ahn et al., 2004). If promptly recognized and treated, the prognosis is usually favorable and the changes, seen in MRI, resolve over days to weeks (Fugate and Rabinstein, 2015).

Many drugs can cause PRES (Abughanimeh et al., 2018; Vilas-Boas and Corte-Real, 2019; Kaur et al., 2020). With new antitumor agents widely used in the clinic in recent years, accumulating case reports have reported the occurrence of PRES in patients receiving targeted therapies, particularly antiangiogenic agents, such as bevacizumab (Hamid et al., 2018; Katada et al., 2018), sunitinib (Saraceno et al., 2017; Rifino et al., 2020), sorafenib (Dogan et al., 2010; Laruelle et al., 2018), pazopanib (Deguchi et al., 2018; Tatsumichi et al., 2021) and regorafenib (Aanes et al., 2018).

From 2006 to 2022, 54 cases of PRES receiving antiangiogenic therapies from the “PubMed” database were reported. Articles were limited to human cases and be published in English. The characteristics of 54 included cases were shown in Table 1. Of the 54 patients, 41 were females and 13 males, and the female-to-male ratio was about 3:1. The median age of patients was 56 years old at the time of diagnosis of PRES. Fifteen patients had a hypertension history and two patients had a diabetes history. Of these patients, 7 cases didn’t develop hypertension and 41 cases developed grade 3 to 4 hypertension according to NCI-CTCAE (version 5.0) during the antiangiogenic therapy. The median time interval from the start of antiangiogenic agents to PRES diagnosis was 33.5 days. As for the patients who had a hypertension history before receiving antiangiogenic therapies, the median time interval from the start of antiangiogenic agents to PRES diagnosis was 21 days, which was earlier than patients without a hypertension history. After discontinuing antiangiogenic therapy and receiving symptomatic treatments, almost all (98.2%) patients achieved complete response, but one patient died.

**TABLE 1:** Cases of PRES associated with antiangiogenic treatment.

Drugs	N	Median age	M:F	Median time (days)	Hypertension	Diabetes	Hypertension (3–4)	Reference No.
Bevacizumab	27	52	5:22	77	5	1	20	Allen et al. (2006), Ozcan et al. (2006), Burki et al. (2008), Koopman et al. (2008), Peter et al. (2008), Dos Reis Simões da Silva et al. (2011), Lau and Paunipagar (2011), Lou et al. (2011), Chang et al. (2012), Lazarus et al. (2012), Sclafani et al. (2012), Seet and Rabinstein (2012), Abbas et al. (2013), Dersch et al. (2013), Goto and Mimura (2014), Sawaya et al. (2014), Wang et al. (2014), Elmalik et al. (2015)
Sunitinib	9	65	2:7	14	3	0	9	Martin et al. (2007), Cumurciuc et al. (2008), Chen and Agarwal (2009), Dos Reis Simões da Silva et al. (2011), Padhy et al. (2011), Khan et al. (2012), Duchnowska et al. (2013), Saraceno et al. (2017)
Pazopanib	7	56	1:6	30	3	0	4	Chelis et al. (2012), Asaithambi et al. (2013), Foerster et al. (2013), Arslan et al. (2017), Miaris et al. (2017), Deguchi et al. (2018), Tatsumichi et al. (2021)
Sorafenib	4	70	1:1	26	3	0	2	Dogan et al. (2010), Furubayashi et al. (2017), Laruelle et al. (2018)
Apatinib	2	52	1:1	51	0	0	1	Li et al. (2018), Lv et al. (2019)
Axitinib	2	58	0:2	8	0	0	2	Levy et al. (2014), Nakamura et al. (2017)
Regorafenib	2	54	1:1	37	0	0	1	Aanes et al. (2018), Van Pelt et al. (2020)
Cediranib	1	65	1	86	1	1	1	Kim et al. (2014)

N, cases, M, male, F, female, median age, the age of diagnosis of PRES, median time, from the start of antiangiogenic agents to PRES diagnosis, hypertension, the number of patients with hypertension history, diabetes, the number of patients with diabetes history, hypertension (3–4), the number of patients developing grade 3 to 4 hypertension during antiangiogenic therapy.

Table 2 summarized the common MRI findings in 54 PRES patients. Thirty-three patients’ imaging findings were symmetrical hemispheric vasogenic edema in the parietal occipital regions, fourteen in basal ganglia, frontal lobe, brainstem, cerebellar hemisphere and unilateral lesion, and seven in the watershed areas of the frontal, parietal, and occipital lobes.

**TABLE 2:** MRI imaging findings in PRES patients.

MRI imaging findings	Cases	Reference no.
Parieto-occipital white matter and cortex were involved, with varying degrees of temporal lobe involvement	33	(Allen et al., 2006; Ozcan et al., 2006; Martin et al., 2007; Burki et al., 2008; Koopman et al., 2008; Peter et al., 2008; Dos Reis Simões da Silva et al., 2011; Lau and Paunipagar, 2011; Lou et al., 2011; Padhy et al., 2011; Chang et al., 2012; Chelis et al., 2012; Khan et al., 2012; Lazarus et al., 2012; Sclafani et al., 2012; Seet and Rabinstein, 2012; Abbas et al., 2013; Asaithambi et al., 2013; Goto and Mimura, 2014; Levy et al., 2014; Sawaya et al., 2014; Eryılmaz et al., 2016; Miller et al., 2016; Nakamura et al., 2017; Saraceno et al., 2017; Aanes et al., 2018; Deguchi et al., 2018; Katada et al., 2018; Li et al., 2018; Rifino et al., 2020; Van Pelt et al., 2020)
Vasogenic edema in the watershed areas of the frontal, parietal, and occipital lobes	7	(Ozcan et al., 2006; Cumurciuc et al., 2008; Koopman et al., 2008; Dersch et al., 2013; Miaris et al., 2017; Katada et al., 2018; Lv et al., 2019)
Basal ganglia, frontal lobe,brainstem, cerebellar hemisphere and unilateral lesion	14	(Koopman et al., 2008; Chen and Agarwal, 2009; Sclafani et al., 2012; Seet and Rabinstein, 2012; Abbas et al., 2013; Dersch et al., 2013; Kim et al., 2014; Elmalik et al., 2015; Arslan et al., 2017; Massey, 2017; Deguchi et al., 2018; Tatsumichi et al., 2021)

Anlotinib demonstrated its efficacy and safety in the ALTER01031 (Li et al., 2021), ALTER0303 (Zhou et al., 2019), and ALTER1202 trials (Cheng et al., 2021), and no PRES was reported. We presented a female patient who contributed to PRES caused by anlotinib. This patient had only hyperglycemia without any other clinical or metabolic derangements and didn’t develop hypertension during the treatment. Of note, after discontinuation of anlotinib, she did not recover from the blurred vision and her brain MRI showed no decrease in cerebral white matter lesions.

The mechanism of PRES induced by antiangiogenic drugs is still unclear. A leading theory of the pathophysiological changes underlying PRES was that rapidly developing hypertension exceeded the upper limit of cerebral blood flow auto-regulation and caused hyper-perfusion (Fugate and Rabinstein, 2015; Parasher and Jhamb, 2020). Based on the literature review, 41 of 54 patients with PRES developed grade 3 to 4 hypertension. Therefore, anti-VEGF therapy-induced hypertension might be a major mechanism of PRES. However, this mechanism could not explain the development of PRES in the 15%–20% of patients who have no hypertension. Additional mechanisms to cause PRES in patients receiving antiangiogenic therapies might exist. Antiangiogenic drugs could lead to vasoconstriction and hypo-perfusion, leading to cerebral ischemia and subsequent vasogenic edema (Morbidelli et al., 2016; Shah, 2017; Gewirtz et al., 2021) (Supplementary Figure 1). Previous studies demonstrated that increased serum pro-inflammatory cytokines including circulating levels of interleukin (IL)-6 and tumor necrosis factor-alpha (TNF-alpha) regulated the expression of vascular endothelial growth factor and they might increase vascular permeability leading to vasogenic edema and cause endothelial damage or dysfunction in PRES (Esposito et al., 2002; Fugate and Rabinstein, 2015). Development of hyperglycemia results in increased serum pro-inflammatory cytokines, such as interleukin (IL)-6 and IL-1b, and C-reactive protein, which stimulates endothelial cells to secrete vasoactive factors and increases vascular permeability (Pickup, 2004; Vaarala and Yki-Järvinen, 2012; Li et al., 2014). Nishanth et al. previously reported one case with hyperglycemia-induced PRES (Dev et al., 2019). From what has been discussed above, hyperglycemia and anlotinib might jointly lead to PRES.

In the reviewed cases, we found that after drug withdrawal almost all (98.2%) patients achieved complete response, which was higher than the incidence (56%–72%) of PRES induced by other causative factors (Bartynski and Boardman, 2007; Legriel et al., 2012). The underlying reversible mechanism was related to reversible dysregulation of the cerebral vasculature (Singhal, 2021), which was completely different from the neurotoxic transformation caused by neurofibrillary tangles in Alzheimer’s disease (Zeng et al., 2022). Higher glycemia on day 1 was a strong predictor of poor outcomes by day 90 after admission for severe PRES in a retrospective cohort study (Legriel et al., 2012). Our case gave the similar outcome, the irreversible changes such as blurred vision. In addition, the patient received WBRT before taking anlotinib. Late radiation-related complications usually occurred more than 6 months after radiotherapy, tended to be irreversible and often progressive (Wujanto et al., 2021). The pathogenesis of late complications was often seen in the white matter and was linked to persistent demyelination, reduced neurogenesis with altered neural stem cell differentiation, inflammatory response through oxidative damage, and disruption of microvasculature resulting in ischaemia and toxic neuro-excitation (Wilke et al., 2018). Thus, hyperglycemia and WBRT was likely to exacerbate the irreversible neurological injury of this case. As we know, once PRES induced by anti-VEGF therapy was confirmed, the therapy should be discontinued. However, how to improve the therapeutic effects for unrecovered patients still requires further investigation.

## Conclusion

Based on our experience and the literature review, the incidence of PRES induced by antiangiogenic drugs including anlotinib is low, and the symptom can resolve upon stopping the medications. However, some cases still have a poor prognosis and the underlying mechanism requires further investigation. In addition, early detection and treatment of PRES are very important for physicians.

## Data Availability

The original contributions presented in the study are included in the article/Supplementary Material, further inquiries can be directed to the corresponding author.
