# Supplementary material for: Posterior reversible encephalopathy syndrome after anlotinib treatment for small cell lung cancer: A case report and literature review
Source: Front Pharmacol. 2023 Feb 6;14:1126235. doi: 10.3389/fphar.2023.1126235 (PMC9939648; doi:10.3389/fphar.2023.1126235)
Supplement: Supplementary file 1 [file Table1.docx]

Supplementary Material

**Posterior Reversible Encephalopathy Syndrome after Anlotinib Treatment for Small Cell Lung Cancer: A Case Report and Literature Review**

**Xiaomeng Zou*, Peng Zhou, Wei Lv, Chuanyong Liu and Jie Liu**

*** Correspondence:**

Jie Liu: sdjnljjie@126.com

Chuanyong Liu: cyl0936@sina.com

1. **Supplementary Figure**

**
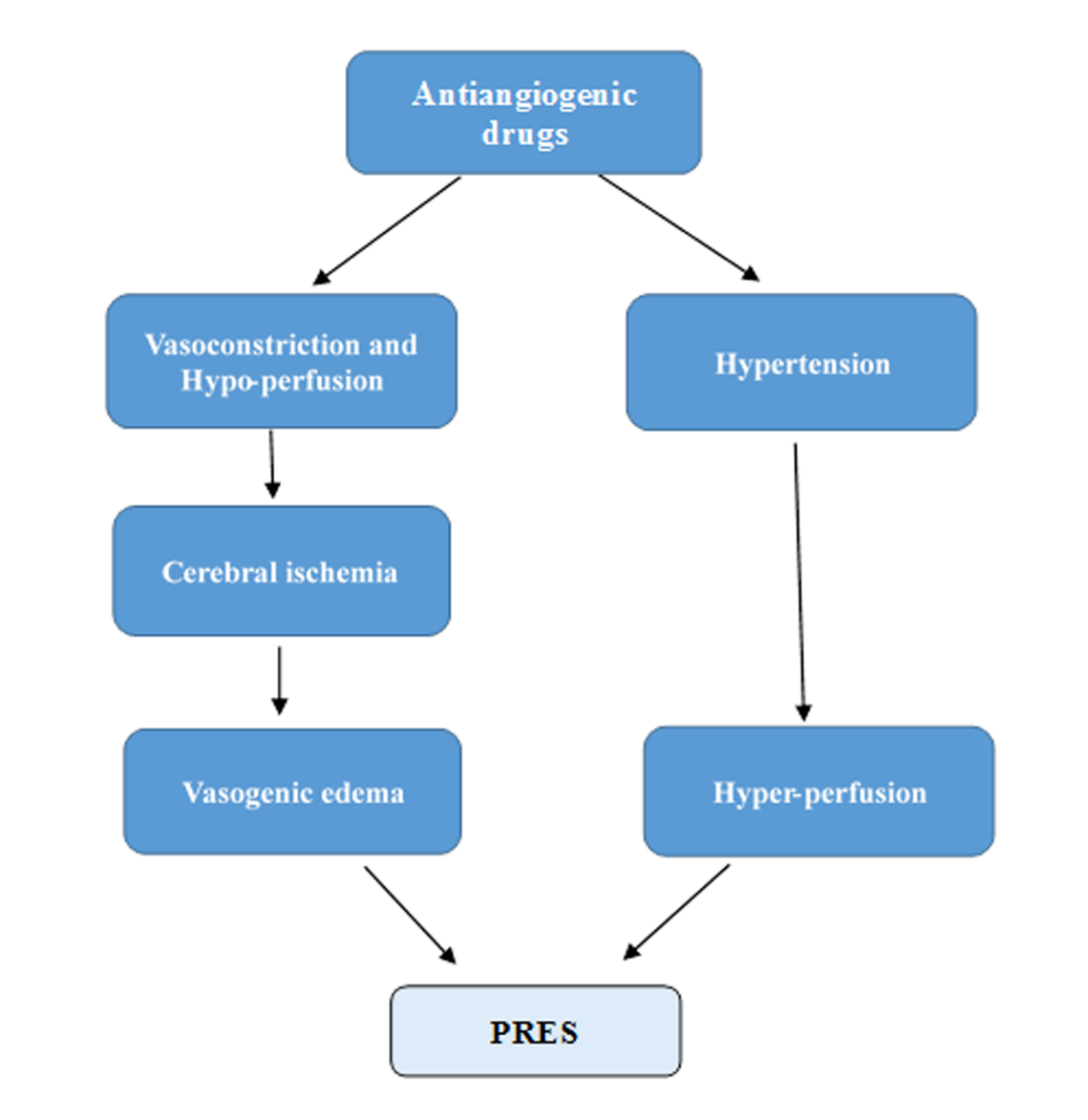
**

**Supplementary Figure 1.** A graphic illustration of the mechanisms for induction of PRES in antiangiogenic treatment.
